# HSPB1 Enhances SIRT2-Mediated G6PD Activation and Promotes Glioma Cell Proliferation

**DOI:** 10.1371/journal.pone.0164285

**Published:** 2016-10-06

**Authors:** Hongxing Ye, Hongguang Huang, Fei Cao, Mantao Chen, Xiujue Zheng, Renya Zhan

**Affiliations:** Department of Neurosurgery, First Affiliated Hospital, School of Medicine, Zhejiang University, Hangzhou, Zhejiang, People’s Republic of China; University of Nebraska-Lincoln, UNITED STATES

## Abstract

Heat shock proteins belong to a conserved protein family and are involved in multiple cellular processes. Heat shock protein 27 (Hsp27), also known as heat HSPB1, participates in cellular responses to not only heat shock, but also oxidative or chemical stresses. However, the contribution of HSPB1 to anti-oxidative response remains unclear. Here, we show that HSPB1 activates G6PD in response to oxidative stress or DNA damage. HSPB1 enhances the binding between G6PD and SIRT2, leading to deacetylation and activation of G6PD. Besides, HSPB1 activates G6PD to sustain cellular NADPH and pentose production in glioma cells. High expression of HSPB1 correlates with poor survivalrate of glioma patients. Together, our study uncovers the molecular mechanism by which HSPB1 activates G6PD to protect cells from oxidative and DNA damage stress.

## Introduction

Heat shock proteins (HSPs) are a group of highly conserved family of proteins[1, 2]. HSPs are highly abundant in mammalian cells, regulating diverse cellular process, such as signal transduction, protein quality control, and stress response[3]. Besides, aberrant expression of HSPs has been reported in multiple human diseases, including renal disease, Alzheimer’s disease, diabetes and cancers, *etc*[4–8].

Heat shock protein 27 (Hsp27), also known as HSPB1, belongs to the small molecular weight heat shock protein family(12-43kDa)[9]. At first, HSPB1 was identified as a protein chaperone that assisted proper refolding of disordered proteins in response to heat shock [10]. Recent evidences suggested that HSPB1 was a multifunction protein, of which the deregulation had been implicated in neuro-degenerative diseases, cardiovascular diseases and cancers, in particular glioma[11–13]. Notably, HSPB1 was expressed at basal level in normal cells[14], whereas glioma cells expressed very high levels of HSPB1[15]. Thus, HSPB1 potentially contributes to the tumorigenesis and development of glioma.

In addition to heat shock response, HSPB1 protected cells from oxidative and chemical stress. Upon exposure to oxidative stress, HSPB1 sustained intracellular glutathione level to scavenge reactive oxygen species (ROS)[16]. Besides, HSPB1 exhibited anti-apoptotic properties in response to toxic chemicals, which ramificated the efficacy of certain DNA damaging agents[17]. Very recently, HSPB1 was reported to activate glucose 6-phosphate dehydrogenase (G6PD), the rate-limiting enzyme in pentose phosphate pathway, in response to oxidative stress [18](.These observations indicate that HSPB1 possibly regulates the metabolism of glioma cells. However, the underlying mechanism of HSPB1-mediated G6PD activation remains unclear. In this study, we aim to investigate how HSPB activates G6PD in response to stressed conditions and its clinical relevance to glioma.

## Materials and Methods

### Antibodies, plasmids, and chemicals

Antibodies against G6PD (Abcam), SIRT2 (Epitomics) were purchased commercially. MNNG (Tokyo Chemical Industry, TCI), temozolomide (TMZ, TCI), H_2_O_2_ (Sigma), diamide (Santa Cruz) were purchased commercially. Catalase expression plasmid was purchased from Addgene.

### Cell culture and treatment

Human glioma cell lines U87-MG and U373-MG, purchased from the American Type Culture Collection (ATCC), were maintained in Dulbecco’s Modified Eagle’s Medium (DMEM) (Invitrogen) supplemented with 10% fetal bovine serum (Biochrom) in the presence of penicillin, streptomycin, and 8 mM L-glutamine (Invitrogen). In TMZ treatment assay, glioma cells were incubated with TMZ 48 h. For MNNG treatment, cells were treated with MNNG for 1 hour, and then cultured in fresh medium for another 47 hours.

### Transfection, immunoprecipitation and western blotting

Cell transfection was performed using Lipofectamine 2000 (Invitrogen) following the manufacturer’s instructions. Cells were lysed in ice-cold NP-40 buffer (50 mM Tris-HCl, pH 7.6, 150 mM NaCl, 0.5% NP-40) containing protease inhibitor cocktail (Roche). Immunoprecipitation was carried out by incubating G6PD or SIRT2 antibody with cell lysate for 1 hr, followed by incubating with Protein-A beads (Upstate) for another two hours at 4°C before beads were washed for three times with ice-cold NP-40 buffer. For western blot, cell lysate was separated on 10% SDS-PAGE and transferred onto PVDF membrane. Membrane was blocked in 5% non-fat milk, followed by incubation with primary antibody overnight at 4°C,and a horseradish peroxidase (HRP)-conjugated secondary antibody for 1hour at room temperature. The membrane was imaged by LAS 4000 (GE healthcare).

### Generation of stable cell pools

Stable cell pools in U87-MG cells were generated as previously described [19]. Briefly, vectors expressing shRNAs against HSPB1 (shHSPB1-A:5’-GGGTCATTGCCATTAATAGAG-3’, shHSPB1-B:5’-GCCATTAATAGAGACCTCAAA-3’) were co-transfected with vectors expressing the *gag* and *vsvg* genes into HEK293T cells using a two-plasmid packaging system as previously described. Retroviral supernatant was harvested 36 hrs after transfection, and mixed with 8 μg/mL polybrene to increase the infection efficiency. Cells were infected with the retrovirus and selected in 1 μg/ml puromycin for 1 week.

### RNAi (RNA interference)

RNAi-mediated G6PD or HSPB1 knockdown was performed by either transfecting control siRNA (small interfering RNA) or siRNAs targeting G6PD or HSPB1 (siG6PD:5’-GCAAACAGAGUGAGCCCUU-3’, siHSPB1:5’-CCAUUAAUAGAGACCUCAA-3’) into U87 or U373 cells following the manufacturer’s instructions (Invitrogen, Lipofectamine RNAiMAX)[20].

### Cell viability assay

For cell viability assay, cells treated with DNA damage reagents or oxidative reagents were collected and stained with propidiumiodide (PI, Sigma) and analyzed by flow cytometry (Beckman Coulter EPICSAltra), or stained with trypanblue (final concentration:0.05–0.1%) for viable cellcounting. MTT assays were performed following the manufacturer’s instruction (Sigma-Aldrich).

### RNA isolation and quantitative real-time PCR

Total RNA was isolated from cultured cells using Trizol reagent (Invitrogen) following the manufacturer’s instructions. RNA was reverse transcribed with oligo-dT primers and preceded to real-time PCR with gene-specific primers in the presence of SYBR Premix Ex Taq (TaKaRa). β-actin was used as a housekeeping control. Sequences of primers for real-time PCR were HSPB1-Forward: 5’-AGCTGACGGTCAAGACCAAG-3’, HSPB1-Reverse:5’-GTGAAGCACCGGGAGATGTA-3’, G6PD-Forward:5’-AGAGCTTTTCCAGGGCGAT-3’, G6PD-Reverse:5’-CACCAGATGGTGGGGTAGAT-3’, 6PGD-Forward:5’-GTCAGTGGTGGAGAGGAAGG-3’, 6PGD-Reverse:5’-GCCTTGGAAGATGGTCTTGA-3’, IDH1-Forward:5’-GTCGTCATGCTTATGGGGAT-3’, IDH1-Reverse:5’-CTTTTGGGTTCCGTCACTTG-3’, ME1-Forward:5’-ACGAATTCATGGAGGCAGTT-3’, ME1-Reverse:5’-GGAGACGAAATGCATTCACA-3’, β-actin-Forward:5’-GCACAGAGCCTCGCCTT-3’, β-actin–Reverse: 5’-GTTGTCGACGACGAGCG-3’.

### Detection of metabolites

Concentrations of nicotinamide adenine dinucleotide phosphate (NADPH/NADP^+^) and glutathione (GSH/GSSG) were determined by using NADP^+^/NADPH Assay Kit (Abcam, #ab65349), and Glutathione Assay Kit (Biovision, #K264-100) following the manufacturer’s instructions.

For Ru-5-P and R-5-P,cellular metabolites were extracted and spectrophotometrically measured. Briefly, in a 3 mL reaction mix, the final concentrations are 58 mM Gly-Gly, 1.7 mM ribose-5-phosphate or 1.7 mM ribulose-5-phosphate, 0.002% (w/v) cocarboxylase, 15 mM MgCl_2_, 0.13 mM β-nicotinamide adenine dinucleotide (NADH), 0.5 units α-glycerophosphate dehydrogenase, 5 units triosephosphate isomerase, 0.5 units transketolase and 0.025–0.05 units D-ribulose-5-phosphate 3-epimerase. Once the reaction was initiated by D-ribulose-5-phosphate 3-epimerase, a decrease in absorbance at 340 nm from NADH oxidation was measured by a DU800 spectrophotometer (Beckman Coulter).

### Detection of reactive oxygen species (ROS)

ROS production was determined by using a fluorescent dye 2’, 7’-dichlorofluorescein diacetate (H_2_DCF-DA, Sigma). Briefly, 4×10^4^ cells were washed with PBS and incubated with 2.5 μM MitoSOX (to measure the mitochondrial ROS superoxide) or 10 μM H_2_DCF-DA at 37°C for 30 min to load the fluorescent dye. Afterward, cells were washed with PBS twice. Fluorescence intensity was monitored by a SpectraMax M5 Microplate Reader (Molecular Devices). Additionally, similar studies were carried using the oxidation-insensitive dye 5-(and -6)-carboxy-2’,7’-dichlorofluorescein diacetate in order to verify that uptake, ester cleavage, and efflux of DCFH-DA dye were not contributing to changes in fluorescence following indicated treatments.

### RNA synthesis

In brief, for the ^14^C-RNA biosynthesis assay, subconfluent cells were spiked with 4 μCi mL^−1^ of D-[U-^14^C] glucose (Perkin Elmer) for 2 hours. Total RNA was extracted using RNeasy columns (Qiagen). The amount of ^14^C-RNA was determined by liquid scintillation counting and normalized by the total amount of RNA. For the ^14^C-lipid biosynthesis assay, lipids were extracted by the addition of 500 μLof hexane/isopropanol (3:2 v/v), dried, resuspended in 50 μLof chloroform, and subjected to scintillation counting.

### Enzyme activity assay

6PGD activity was determined on the basis of the rate of NADPH production in assay buffer containing 0.1 mM NADP^+^, 1 mM MgCl_2_ and 50 mM Tris (pH 8.1) with 0.2 mM 6-phosphogluconate as a substrate. The increase of absorbance at 340 nm was measured by a spectrophotometer.

G6PD activity was determined by the NADPH production rate from G6PD and 6PGD, and then subtracting that of 6PGD, because a product of G6PD, 6-phosphogluconolactone, is rapidly hydrolysed to a substrate of 6PGD, 6-phosphogluconate, in cells.

IDH1 activity was measured by using cytosolic extracts.The reaction mixture contains 20 mM Gly-Gly (pH 7.5), 0.6 mM MnCl_2_, 1 mM NADP^+^ and 0.44 mM D-(+)-threo-isocitrate. Increase in 340 nm absorbance as a measure of NADPH production was detected every 20 s for 10 min on a DU800 Spectrophotometer (Beckman Coulter).

The reaction buffer of ME1 activity assay contained 67 mM triethanolamine, 3.3 mM l-malic acid, 0.3 mM NADP^+^ and 5.0 mM manganese chloride.The reactions were started by adding cytosolic extracts and were monitored by absorbance at 340 nm every 5 s for up to 10 min. Background control was run without l-malic acid as substrate.

### Statistical analyses

Statistical analyses were performed with a two-tailed unpaired Student's t-test. All data shown represent the results obtained from triplicated independent experiments with standard errors of the mean (mean ± S.D.). Kaplan-Meier survival curves were prepared using tumors with mRNA data from TCGA. The patients were stratified by median mRNA expression Z-score. ‘High’ indicates greater than median, ‘low’ denotes less than or equal to median. P-value was determined by the log-rank test, values of p<0.05 were considered statistically significant.

## Results

### HSPB1 contributes to cell proliferation and survival of U87 MG glioma cells

**Fig 1:**
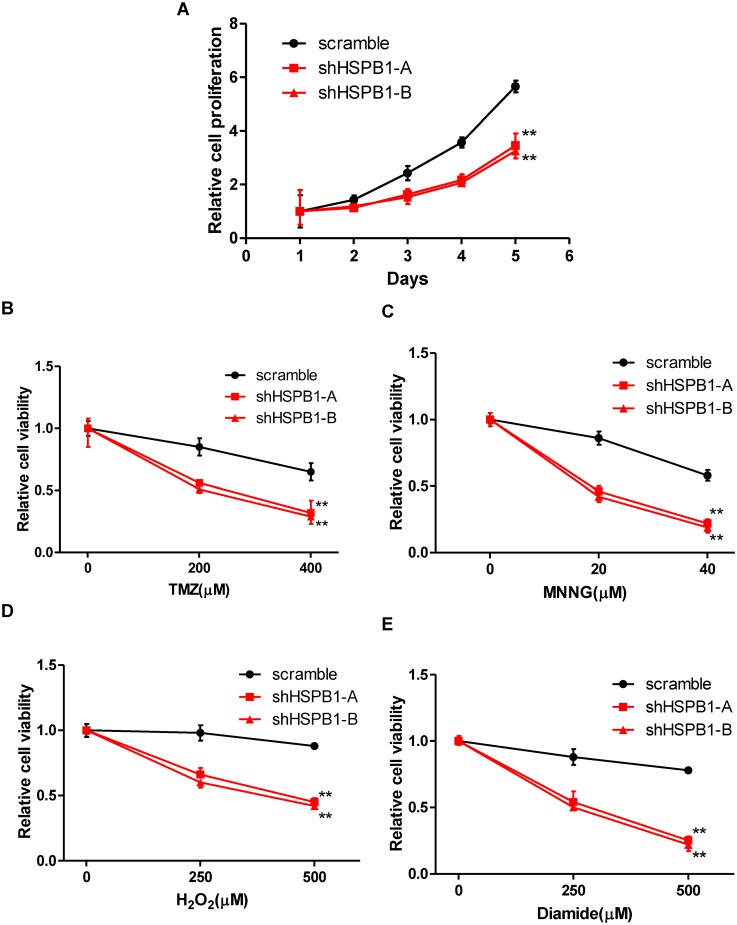
HSPB1 contributes to cell proliferation and survival of U87-MG glioma cells. (A) Cell proliferation of U87-MG cells expressing shRNAs against HSPB1 was determined. (B-C) HSPB1-knockdown stable U87 cells were treated with increasing concentrations of TMZ or MNNG, cell viability was determined.(D-E) HSPB1-knockdown stable U87 cells were treated with increasing concentrations of H_2_O_2_ or diamide, cell viability was determined.

To determine the role of HSPB1 in the proliferation and stress responseof glioma cells, we established stable U87 glioma cells expressing shRNAs against HSPB1 (S1 Fig). Interestingly, knockdown of HSPB1 led to a significant reduction in the proliferation of U87 cells (Fig 1A). This result suggests that HSPB1 is a crucial factor for the growth of glioma cells. HSPB1 might function as a chaperone and protect glioma cells from stressed conditions. To test this hypothesis, we determined the cell viability of U87 stable cells after exposure to DNA damaging reagents or oxidative reagents. Interestingly, treatment of temozolomide (TMZ) and N-methyl-N'-nitro-N-nitrosoguanidine(MNNG), two DNA alkylating agents, led to more pronounced cell death in HSPB1-deficient cells, but not control U87 MG cells, in a dose-dependent manner (Fig 1B and 1C). Moreover, HSPB1-knockdown stable cells were more vulnerable to oxidative reagents, i.e. H_2_O_2_ and diamide (Fig 1D and 1E), than cells expressing control shRNA. These observations demonstrate that HSPB1 contributes to the proliferation and stress response of glioma cells. The protective role of HSPB1 also suggests potential involvement of HSPB1 in chemo-resistance of glioma cells.

### HSPB1 sustains cellular NADPH and pentose levels

**Fig 2:**
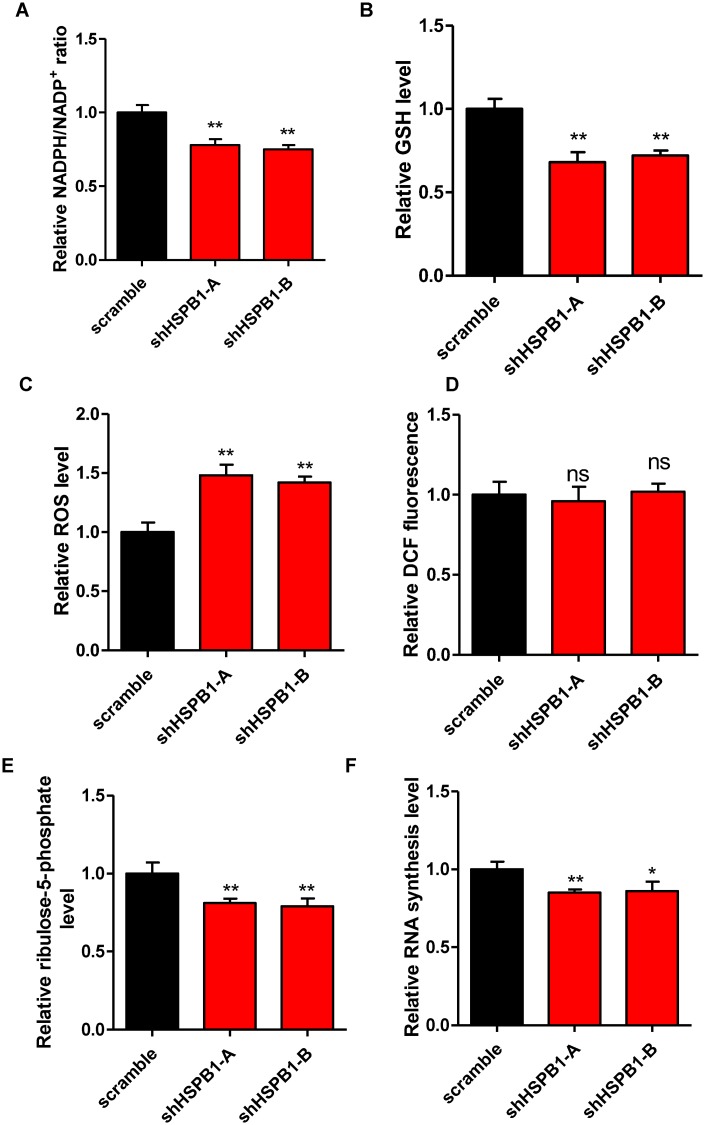
HSPB1 sustains cellular NADPH and pentose levels. (A) NADPH/NADP^+^ ratio in stable HSPB1-knockdown U87 cells was determined. (B) GSH level in stable HSPB1-knockdown U87 cellswas quantified. (C-D) ROS level in stable HSPB1-knockdown U87 cells was determined by using DCF (C). An oxidation-insensitive dye was included as a negative control (D).(E) Ribulose-5-phosphate content in stable HSPB1-knockdown U87 cells was quantified. (F) Relative RNA synthesis rate in stable HSPB1-knockdown U87 cells was determined.

To identify potential cellular processes regulated by HSPB1, we tested the effect of HSPB1 depletion on DNA damage repair and ROS buffering system. Knockdown of HSPB1 led to a 22% reduction of NADPH/NADP^+^ ratio in U87 cells (Fig 2A). Besides, glutathione (GSH, a reducing metabolite) level was significantly down-regulated by HSPB1 knockdown (Fig 2B), with a corresponding 45% increase of cellular ROS level (Fig 2C). As a negative control, the fluorescence of an oxidation-insensitive dye remained unchanged by HSPB1 knockdown (Fig 2D), confirming that depletion of HSPB1 indeed resulted in ROS increase. Meanwhile, overexpression of catalase, a primary anti-oxidant enzyme, reduced cellular ROS, further demonstrating that depletion of HSPB1 exaggerated oxidative stress (S2 Fig). We also determined the mitochondria ROS level by using MitoSOX reagent and observed a negligible increase of mitochondrial ROS (S3 Fig). These results indicate that HSPB1 regulates ROS buffering system in the cytoplasm. Additionally, cytosolic pentose pool provides precursors for DNA repair reactions. To determine the effect of HSPB1 on cellular pentose synthesis, we carried out liquid chromatography–mass spectrometry (LC-MS) analysis and found that depletion of HSPB1 led to a 20% reduction of cellular ribulose-5-phosphate (R5P) level (Fig 2E). This result suggests that HSPB1 knockdown results in insufficient pentose supply, supporting the notion that HSPB1-depleting cells were more vulnerable to DNA damage reagents. Of note, shortage of pentose might also impair certain biosynthetic processes, such as RNA biosynthesis. As expected, RNA synthesis was suppressedby 15% in HSPB1-knockdown stable cells, compared to control U87 cells (Fig 2F). These data suggest that HSPB1 functions in maintaining cellular NADPH and pentose levels, which further modulates ROS scavenging and DNA repair processes.

### HSPB1 sustains G6PD activity

**Fig 3:**
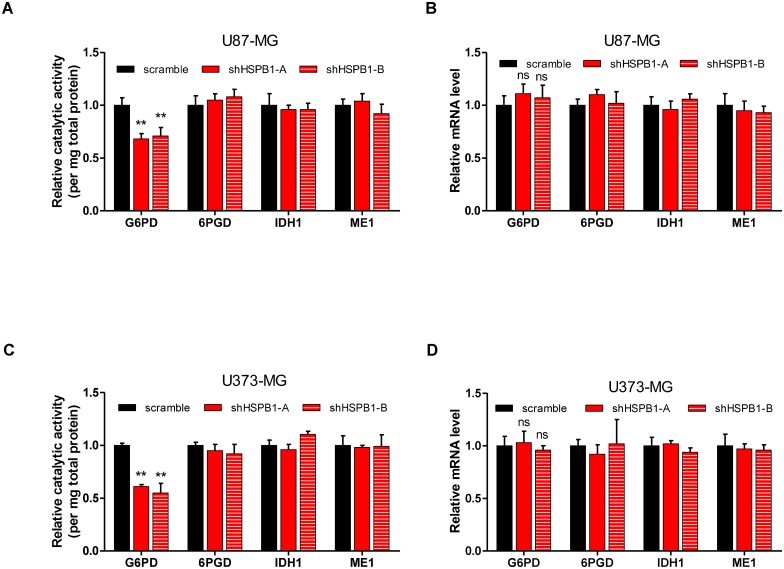
HSPB1 sustains G6PD activity. (A) Relative activity of G6PD, 6PGD, IDH1 and ME1 was assayed in control or HSPB1-knockdown U87 cells.(B) Relative mRNA expression of G6PD, 6PGD, IDH1 and ME1 was determined in control or HSPB1-knockdown U87 cells.(C) Relative activity of G6PD, 6PGD, IDH1 and ME1 was determined in control or HSPB1-knockdown U373 cells.(D) Relative mRNA expression of G6PD, 6PGD, IDH1 and ME1 was quantified in control or HSPB1-knockdown U373 cells.

The observation that HSPB1 knockdown increased cellular ROS level without interfering mitochondria ROS led us to speculate that HSPB1 regulated cytosolic ROS buffering system. Cellular redox homeostasis was tightly correlated with NADPH, an important reducing power. Thus, HSPB1 probably sustained cellular NADPH level through maintaining the activity of NADPH-producing enzymes. To this end, we determined the catalytic activities of four cytosolic NADPH producing enzymes, i.e. G6PD, 6-phosphogluconate dehydrogenase(6PGD), malic enzyme 1 (ME1) and isocitrate dehydrogenase 1 (IDH1)[21]. Interestingly, HSPB1 knockdown reduced the activity of G6PD, but not other NADPH producing enzymes (Fig 3A). Besides, the mRNA expression of these enzymes remained unchanged by HSPB1 knockdown (Fig 3B). Similar results were achieved from U373-MG glioma cells (Fig 3C and 3D). Together, these data indicate that HSPB1 sustains G6PD activity in glioma cells.

### HSPB1 activates G6PD by promoting its deacetylation

**Fig 4:**
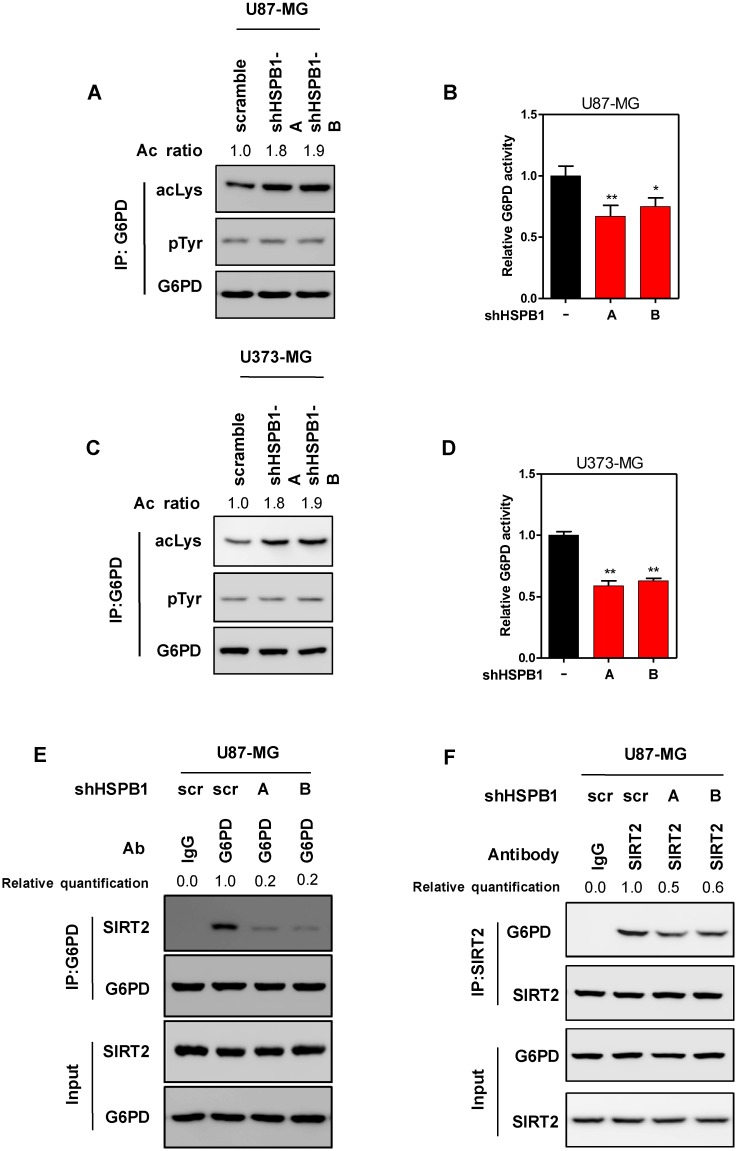
HSPB1 activates G6PD by promoting its SIRT2 interaction and deacetylation. (A) Acetylation and phosphorylation levels of immunoprecipitated endogenous G6PD from HSPB1-knockdown U87 cells were determined by western blot. (B) The activity of endogenous G6PD was determined in HSPB1-knockdown U87 cells. (C) Acetylation and phosphorylation level of immunoprecipitated endogenous G6PD from HSPB1-knockdown U373 cells was determined by western blot. (D) The activity of endogenous G6PD was determined in HSPB1-knockdown U373 cells. (E) Interaction between endogenous G6PD and SIRT2 was determined by immunoprecipitation and western blot in HSPB1-knockdown U87 cells.(F) Interaction between endogenous G6PD and SIRT2 was determined by an inverse co-immunoprecipitation and western blot in HSPB1-knockdown U87 cells.

Previous studies found that HSPB1/Hsp27 enhanced G6PD activity under irradiation [18]. However, the underlying mechanism of G6PD activation remained unclear. As the mRNA expression of G6PD was unaffected by HSPB1 knockdown, we speculated that loss of HSPB1 might affect the post-translational modification of G6PD. G6PD was identified to be modified by phosphorylation and acetylation. Interestingly, phosphorylation level of G6PD was not changed by HSPB1 knockdown, while its acetylation level was remarkably increased by HSPB1 depletion (Fig 4A). In accord, G6PD activity was significantly decreased in HSPB1-knockdown U87 cells (Fig 4B). Depleting HSPB1 in U373-MG resulted in consistent effects (Fig 4C and 4D). These results suggest that HSPB1 is involved in the acetylation regulation of G6PD. Protein deacetylase SIRT2 had been identified as the principle regulator of G6PD acetylation [22].It is possible that HSPB1 modulatesG6PD acetylation through altering protein-protein interaction. As expected, the binding of G6PD and SIRT2 was severely impaired in HSPB1-knockdown cells (Fig 4E). This result was further confirmed by an inverse co-immunoprecipitation of SIRT2 and G6PD (Fig 4F). Taken together, HSPB1 promotes the physical interaction between SIRT2 and G6PD, which further induces deacetylation and activation of G6PD.

### Inhibiting HSPB1-mediated G6PD activation reduces glioma cell survival under DNA damage and oxidative stress

**Fig 5:**
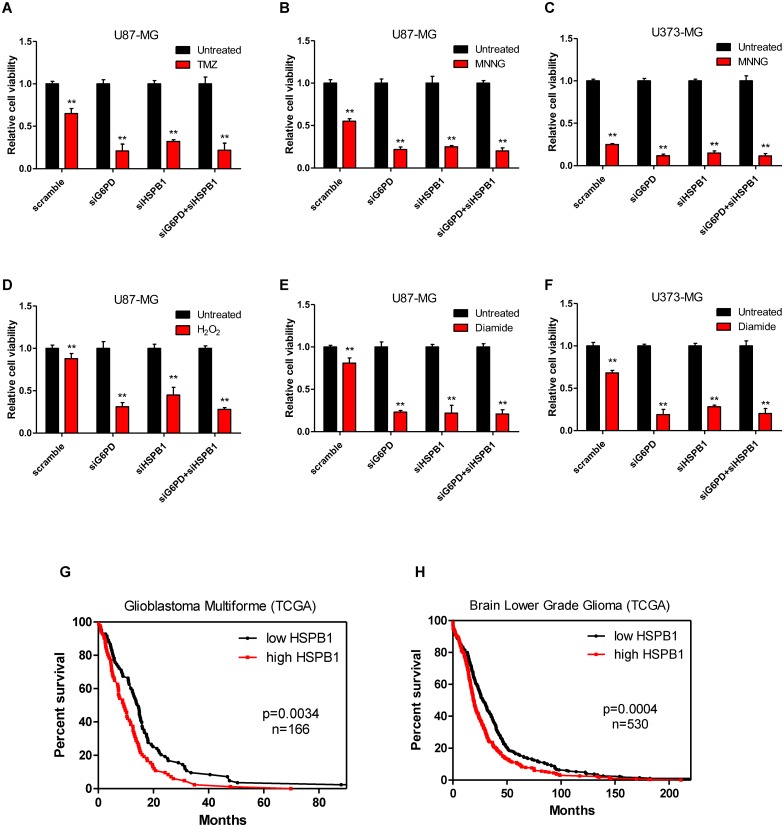
Inhibiting HSPB1-mediated G6PD activation attenuates the chemo-resistance of glioma cells. (A-B) U87 cells were transfected with siRNAs against G6PD and/or HSPB1 as indicated. Cells were treated with TMZ (A) or MNNG (B), cell viability was determined. (C) U373 cells were transfected with siRNAs against G6PD and/or HSPB1 as indicated. Cells were treated with MNNG, cell viability was determined.(D-E) U87 cells were transfected with siRNAs against G6PD and/or HSPB1 as indicated. Cells were treated with H_2_O_2_ (D) or diamide (E), cell viability was determined. (F) U373 cells were transfected with siRNAs against G6PD and/or HSPB1 as indicated. Cells were treated with diamide, cell viability was determined. (G-H) Kaplan-Meier survival curves for TCGA glioblastoma multiforme (G) and brain lower grade glioma (H) study. The patients were stratified by median HSPB1 levels (‘high’ is greater than median, ‘low’ is less than or equal to median).

To explore the functional significance of HSPB1-mediated G6PD activation during stress response, we depleted HSPB1 and/or G6PD using siRNAs in U87 and U373 cells (S4 Fig). Knockdownof either G6PD or HSPB1 led to increased cell death when cells were exposed to TMZ or MNNG. Meanwhile, no additive effect was observed when both G6PD and HSPB1 were depleted (Fig 5A, 5B and 5C). Similarly, knockdown of either G6PD or HSPB1 sensitized glioma cells to oxidative reagents (H_2_O_2_ or diamide), but no additive effect was found in HSPB1- and G6PD-knockdown cells (Fig 5D, 5E and 5F). These data suggest that G6PD is a major target in HSPB1-sustained cells survival under DNA damage or ROS stress. Additionally, we investigated two TCGA cohorts (glioblastoma and brain low-grade glioma) and found that high HSPB1 expression correlated with poor overall survival rate (p = 0.0034 for TCGA glioblastoma multiforme; p = 0.0004 for TCGA Brain Low Grade Glioma) (Fig 5G and 5H). These findings strongly support the notion that HSPB1 contributes to the proliferation and survival of glioma cells.

**Fig 6:**
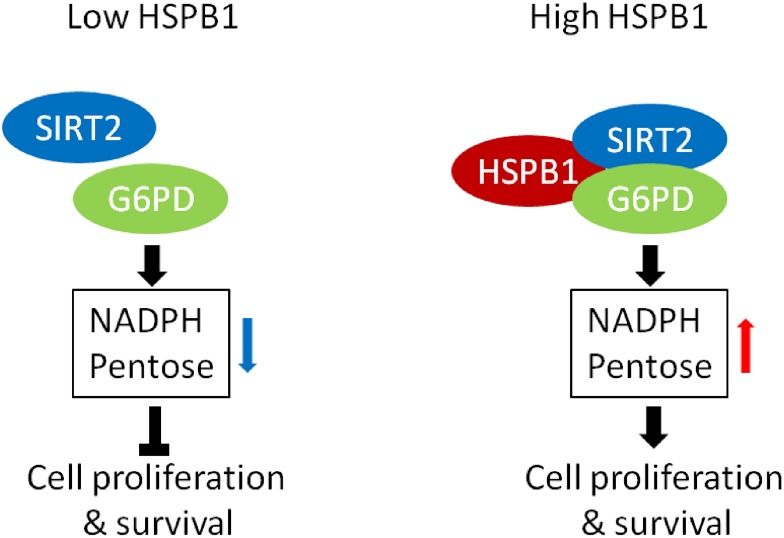
The working model for HSPB1 mediated G6PD activation.

In conclusion, our study revealed that HSPB1-mediated G6PD activation sustained cellular NADPH and pentose levels, and promoted glioma cell proliferation and survival (Fig 6).

## Discussion

Previous studies suggested a protective role of HSPB1 under oxidative and chemical stress. However, the underlying mechanism was poorly understood. In this study, we demonstrated that HSPB1 sustained cellular NADPH and pentose production in glioma cells. HSPB1 activatedG6PD, the first and rate-limiting enzyme of pentose phosphate pathway, through enhancing SIRT2-mediated deacetylation of G6PD, hence protected glioma cells from oxidative stress and DNA damage.

HSPB1 overexpression was considered to impair the efficiency of chemotherapy in glioma [23]. Thus, suppression of HSPB1 possibly sensitizes glioma cells to alkylating reagents. Here, we found that HSPB1 facilitated SIRT2-mediated deacetylation and activation of G6PD to support NADPH and pentose synthesis. These observations indicate that the tumor-promoting role of HSPB1 is at least in part dependent on G6PD. Besides, it is probable that combined treatment of glioma with SIRT2 or G6PD inhibitor would enhance anti-cancer effect of alkylating agent. Further in vivo or clinical studies are required to investigate this potential strategy of suppressing glioma.

Clinically, HSPB1 overexpression correlated with poor survival rate in both glioblastoma and brain low-grade glioma. This observation suggests potential prognostic value of HSPB1 expression. As HSPB1 contributes to glioma cell growth and survival through regulating SIRT2-G6PD interaction, the relationship between HSPB1 expression and the development of glioma, merits further explorations.

## Conclusions

HSPB1 activates G6PD by promoting SIRT2-mediated G6PD deacetylation, thereby contributing to glioma cell survival and proliferation.

## Supporting Information

S1 Fig Knockdown efficiency of shRNA targeting HSPB1. Knockdown efficiency of shRNAs targeting HSPB1 in U87 MG was determined by qPCR.
(PDF)

S2 Fig Overexpression of catalase decreases cellular ROS level. Flag-tagged catalase was overexpressed in U87-MG cells, cellular ROS level was determined.
(PDF)

S3 Fig Mitochondria ROS level in HSPB1-knockdown cells. Mitochondria ROS level in U87 cells stably expressing shRNAs against HSPB1 was determined using MitoSox.
(PDF)

S4 Fig Knockdown efficiency of shRNAs targeting HSPB1 or G6PD. siRNAs against G6PD and/or HSPB1 was transfected into U87 cells (upper) and U373 cells (lower) as indicated. Knockdown efficiency was determined by qPCR.
(PDF)
