# Supplementary material for: HSPB1 Enhances SIRT2-Mediated G6PD Activation and Promotes Glioma Cell Proliferation
Source: PLoS One. 2016 Oct 6;11(10):e0164285. doi: 10.1371/journal.pone.0164285 (PMC5053603; doi:10.1371/journal.pone.0164285)
Supplement: S1 Fig — Knockdown efficiency of shRNAs targeting HSPB1 in U87 MG was determined by qPCR. (PDF) [file pone.0164285.s001.pdf]

S1 Fig. Knockdown efficiency of shRNA targeting HSPB1

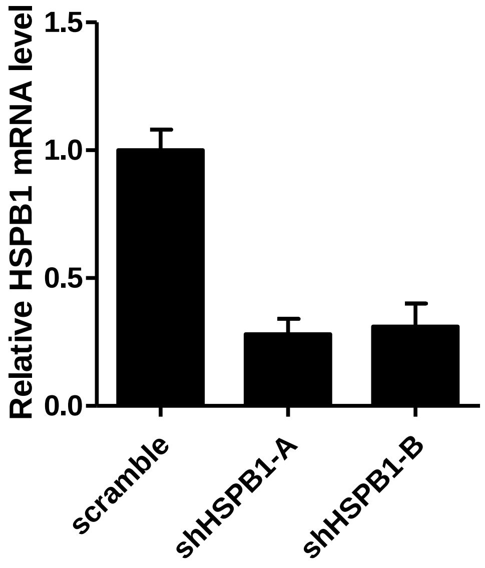

S1 Fig. Knockdown efficiency of shRNAs targeting HSPB1 in U87 MG was determined by qPCR.
