# Supplementary material for: HSPB1 Enhances SIRT2-Mediated G6PD Activation and Promotes Glioma Cell Proliferation
Source: PLoS One. 2016 Oct 6;11(10):e0164285. doi: 10.1371/journal.pone.0164285 (PMC5053603; doi:10.1371/journal.pone.0164285)
Supplement: S2 Fig — Flag-tagged catalase was overexpressed in U87-MG cells, cellular ROS level was determined. (PDF) [file pone.0164285.s002.pdf]

S2 Fig. Overexpression of catalase decreases cellular ROS level

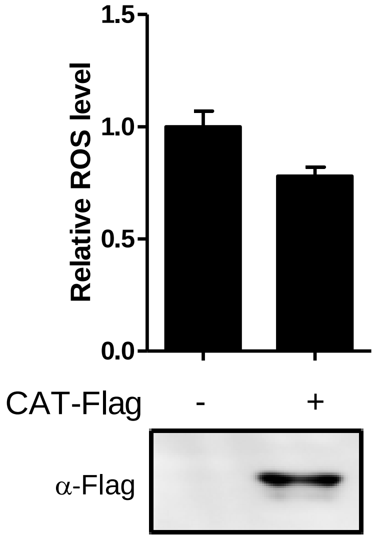

S2 Fig. Flag-tagged catalase was overexpressed in U87-MG cells, cellular ROS level was determined
