# Supplementary material for: HSPB1 Enhances SIRT2-Mediated G6PD Activation and Promotes Glioma Cell Proliferation
Source: PLoS One. 2016 Oct 6;11(10):e0164285. doi: 10.1371/journal.pone.0164285 (PMC5053603; doi:10.1371/journal.pone.0164285)
Supplement: S3 Fig — Mitochondria ROS level in U87 cells stably expressing shRNAs against HSPB1 was determined using MitoSox. (PDF) [file pone.0164285.s003.pdf]

S3 Fig. Mitochondria ROS level in HSPB1-knockdown cells

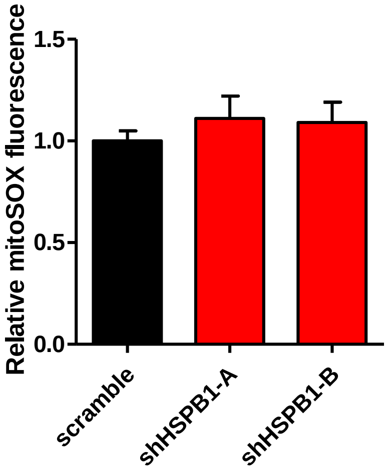

S3 Fig. Mitochondria ROS level in U87 cells stably expressing shRNAs against HSPB1 was determined using MitoSox.
