# Supplementary material for: HSPB1 Enhances SIRT2-Mediated G6PD Activation and Promotes Glioma Cell Proliferation
Source: PLoS One. 2016 Oct 6;11(10):e0164285. doi: 10.1371/journal.pone.0164285 (PMC5053603; doi:10.1371/journal.pone.0164285)
Supplement: S4 Fig — siRNAs against G6PD and/or HSPB1 was transfected into U87 cells (upper) and U373 cells (lower) as indicated. Knockdown efficiency was determined by qPCR. (PDF) [file pone.0164285.s004.pdf]

S4 Fig. Knockdown efficiency of shRNAs targeting HSPB1 or G6PD

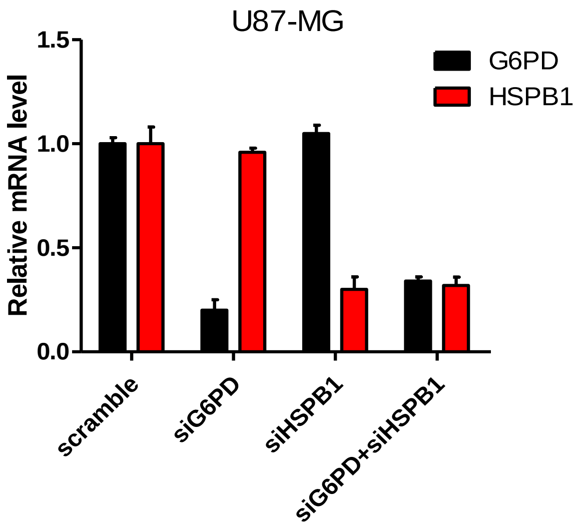

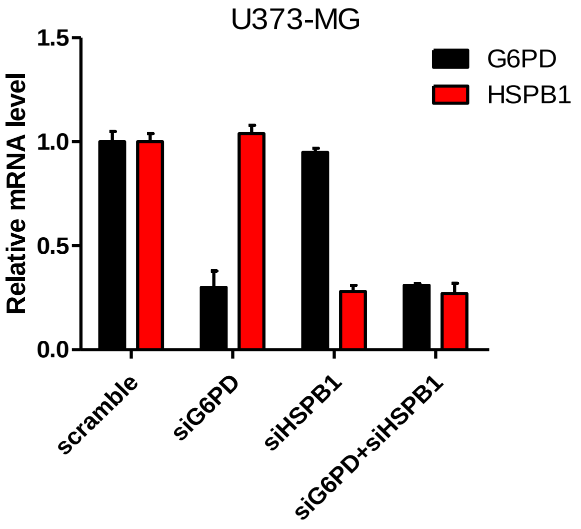

S4 Fig. siRNAs against G6PD and/or HSPB1 was transfected into U87 cells (upper) and U373 cells (lower) as indicated. Knockdown efficiency was determined by qPCR.
